# On the relationship between anxiety and error monitoring: a meta-analysis and conceptual framework

**DOI:** 10.3389/fnhum.2013.00466

**Published:** 2013-08-15

**Authors:** Jason S. Moser, Tim P. Moran, Hans S. Schroder, M. Brent Donnellan, Nick Yeung

**Affiliations:** ^1^Department of Psychology, Michigan State UniversityEast Lansing, MI, USA; ^2^Department of Psychology, University of OxfordOxford, UK

**Keywords:** anxiety, error monitoring, error-related negativity, conflict monitoring, cognitive control, event-related potential (ERP), meta-analysis, worry

## Abstract

Research involving event-related brain potentials has revealed that anxiety is associated with enhanced error monitoring, as reflected in increased amplitude of the error-related negativity (ERN). The nature of the relationship between anxiety and error monitoring is unclear, however. Through meta-analysis and a critical review of the literature, we argue that anxious apprehension/worry is the dimension of anxiety most closely associated with error monitoring. Although, overall, anxiety demonstrated a robust, “small-to-medium” relationship with enhanced ERN (*r* = −0.25), studies employing measures of anxious apprehension show a threefold greater effect size estimate (*r* = −0.35) than those utilizing other measures of anxiety (*r* = −0.09). Our conceptual framework helps explain this more specific relationship between anxiety and enhanced ERN and delineates the unique roles of worry, conflict processing, and modes of cognitive control. Collectively, our analysis suggests that enhanced ERN in anxiety results from the interplay of a decrease in processes supporting active goal maintenance and a compensatory increase in processes dedicated to transient reactivation of task goals on an as-needed basis when salient events (i.e., errors) occur.

## Introduction

Anxiety is a common human experience characterized by a variety of symptoms, including worrisome thoughts, physiologic arousal, and strategic avoidance behaviors (Barlow, 2002). It generally serves an adaptive response to threat by motivating organisms to increase their vigilance and thus respond more effectively to threats (Marks and Nesse, 1994; Barlow, 2002). Excessive and persistent anxiety, however, represents one of the most prevalent mental health problems in the United States (Kessler et al., 2005, 2012; Kroenke et al., 2007) and elsewhere (e.g., Collins et al., 2011 for a review). Research from diverse literatures indicates that cognitive deficits represent a core aspect of the pathological anxiety that is associated with impairments in personal functioning (American Psychiatric Association, 2000; Eysenck et al., 2007; Beilock, 2008; Sylvester et al., 2012). Better understanding the associations between anxiety and cognitive deficits is therefore of great importance for helping to address problems stemming from pathological anxiety.

One especially active area of neuroscience research aimed at tackling this issue has focused on how anxiety is related to error monitoring. Error monitoring concerns the signaling and detection of errors in order to optimize behavior across a range of tasks and situations, and this monitoring function is therefore a fundamental component of behavioral regulation. A growing body of research indicates that anxiety is associated with enhanced amplitude of the error-related negativity (ERN) of the human event-related brain potential (ERP), suggesting that anxiety is associated with exaggerated error monitoring (Olvet and Hajcak, 2008).

Anxiety is not a monolithic construct, however. Researchers and laypersons alike use the term “anxiety” to refer to many different states and traits such as “stress,” “fear,” “worry,” among others (cf. Barlow, 2002). This confusion contributes to difficulties with describing the *nature* of the relationship anxiety has with error monitoring, and the ERN, more specifically. Nonetheless, many agree that there is a useful distinction between anxious apprehension on the one hand and anxious arousal on the other (Nitschke et al., 2001; Barlow, 2002). Anxious apprehension is defined by worry and verbal rumination elicited by ambiguous future threats whereas anxious arousal is defined by somatic tension and physiological hyperarousal elicited by clear and present threats. We and others have recently suggested that the ERN is more closely associated with anxious apprehension than anxious arousal (Moser et al., 2012; Vaidyanathan et al., 2012; Weinberg et al., 2012b).

The purpose of the current review is to expand on this argument in two important ways: (1) by conducting the first large-scale test of this hypothesis using meta-analysis, and (2) by providing a detailed conceptual framework that can be used to generate mechanistic hypotheses and guide future studies. Regarding the latter, we leverage four key findings about anxiety and cognitive control: (1) anxious apprehension/worry is significantly involved in cognitive abnormalities in anxiety; (2) anxious performance is characterized by processing inefficiency; (3) enhanced ERN in anxiety is observed without corresponding deficits in task performance; and (4) individuals with chronic anxiety exhibit enhanced transient “reactive” control but reduced preparatory “proactive” control. We used these findings to develop a new *compensatory error monitoring* account of enhanced ERN in anxiety. Specifically, we suggest that the enhanced ERN observed in anxiety results from the interplay of a decrease in processes supporting active goal maintenance, because of the distracting effects of worry, and a compensatory increase in processes dedicated to transient reactivation of task goals on an as-needed basis when salient events (i.e., errors) occur. The overall format of this integrative review follows that of others in the literature by incorporating both empirical and theoretical considerations throughout the narrative (e.g., Holroyd and Coles, 2002; Shackman et al., 2011; Yeung et al., 2004).

### The error-related negativity (ERN)

**Figure 1:**
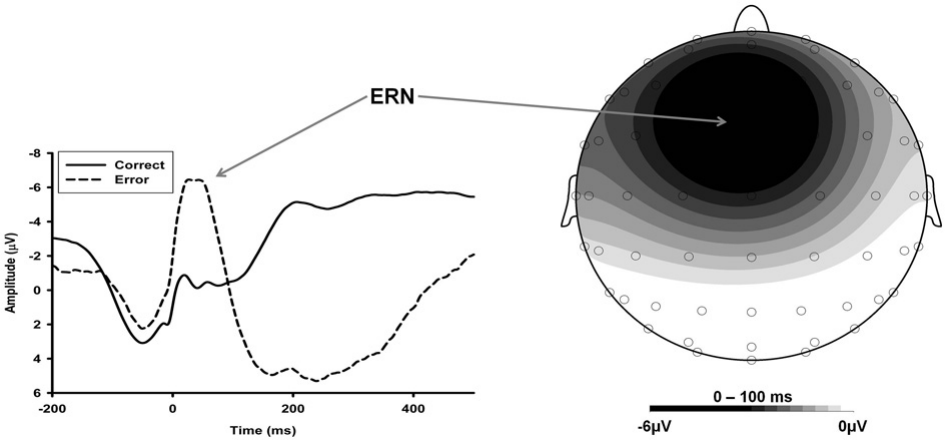
**ERN Waveform and Voltage Map**. Neural activity recorded in the post-response period during a flanker task. Response-locked waveform is presented on the left. Dashed line: the ERN is shown as the negative deflection peaking at approximately 50 ms; the ERN is followed by a broad, positive deflection—the error-positivity. Solid line: the CRN is the correct-response counterpart to the ERN. It shows a similar time course and scalp distribution. A voltage map depicting the scalp distribution of the ERN is presented on the right. It shows that the ERN is primarily a fronto-centrally maximal negativity.

The ERN is an ERP component that reaches maximal amplitude over frontocentral recording sites within 100 ms after response errors in simple reaction time tasks (See Figure 1; Falkenstein et al., 1991; Gehring et al., 1993; see Gehring et al., 2012 for a review). Converging evidence suggests the anterior cingulate cortex (ACC) is involved in the generation of the ERN. More specifically, the dorsal portion of the ACC (dACC) or midcingulate cortex (MCC; Shackman et al., 2011) appears particularly important to the generation of the ERN (Gehring et al., 2012). The dACC/MCC has neuronal projections extending to motor cortex, lateral prefrontal cortex, parietal cortex, basal ganglia, and emotional centers such as the amygdala, suggesting that it serves as a “central hub” in which cognitive and emotional information is integrated and utilized to adaptively adjust behavior (Shackman et al., 2011). It is important, however, to distinguish between the ERN and dACC/MCC activity, as the ERN is a scalp-recorded potential that has several possible sources in other regions of cortex, including lateral prefrontal, orbitofrontal, and motor cortices (Gehring et al., 2012).

The confluence of cognitive and emotional processing within the dACC/MCC has contributed to disagreements among researchers regarding the functional significance of the ERN. To date, however, the two dominant models of the function significance of the ERN are the conflict monitoring (Yeung et al., 2004) and reinforcement learning (Holroyd and Coles, 2002) theories. The conflict monitoring theory suggests the ERN reflects detection by dACC/MCC of the co-activation of mutually exclusive response tendencies; the erroneous response and the subsequent error-correcting response activated immediately after error onset (Yeung and Cohen, 2006). The reinforcement learning theory suggests the ERN reflects the impact on dACC/MCC of a phasic dip in midbrain dopamine release whenever outcomes are worse than expected. This mechanism ultimately trains the dACC/MCC to maximize performance on the task at hand (Holroyd and Coles, 2002). These theories have both garnered support in the literature, and more inclusive “second generation” models have been proposed to incorporate both conflict monitoring and reinforcement learning aspects (Alexander and Brown, 2011; Holroyd and Yeung, 2012).

### The ERN and anxiety

Numerous studies have noted that individual differences in anxiety are associated with increased ERN amplitude (for reviews, see Olvet and Hajcak, 2008; Simons, 2010; Vaidyanathan et al., 2012; Weinberg et al., 2012b). The most robust evidence emerges from research on symptoms and categorical diagnoses of generalized anxiety disorder (GAD; Hajcak et al., 2003; Weinberg et al., 2010, 2012a) and obsessive-compulsive disorder (OCD; see Mathews et al., 2012 for a review)^1^. Because GAD and OCD are largely characterized by worry and verbal rumination (American Psychiatric Association, 2000; Barlow, 2002), we suggested that this work is consistent with our thesis that the ERN is most closely associated with anxious apprehension. Indeed, we directly showed that the ERN was more strongly related to a measure of anxious apprehension than a measure of anxious arousal in a sample of female undergraduates (Moser et al., 2012). Hajcak et al. (2003) demonstrated a similar effect such that the ERN was enhanced in college students high in anxious apprehension but not in students highly phobic of spiders. Other recent descriptive reviews of the literature have come to a similar conclusion that the ERN is aligned most consistently with anxious apprehension (Vaidyanathan et al., 2012; Weinberg et al., 2012b).

### Aims of the current meta-analysis

Despite evidence pointing to a specific association between anxious apprehension and enhanced ERN, very few empirical demonstrations of this specificity have been conducted. We aimed to address this gap by employing meta-analysis to provide a large-scale test of the hypothesis that anxious apprehension is the dimension of anxiety most closely associated with enhanced ERN.

Although our main focus for the meta-analysis is on the ERN, we also report findings related to the correct-response negativity (CRN). The CRN is a negative ERP component observed following correct responses that has similar topography, morphology, and perhaps functional significance to the ERN (See Figure 1; Vidal et al., 2000, 2003; Bartholow et al., 2005). Some studies have reported that anxiety is associated with enhancement in overall negativity following responses, including both the ERN and CRN, suggesting overactive response monitoring in general (Hajcak and Simons, 2002; Hajcak et al., 2004; Endrass et al., 2008, 2010; Moser et al., 2012). Thus, it is important to investigate how anxiety is related to the CRN. Moreover, to isolate error-specific activity from correct-related activity, we examined the relationship between anxiety and the difference between the ERN and CRN—i.e., the ΔERN (see Weinberg et al., 2010, 2012a).

## Materials and methods

### Study selection

Published studies examining the ERN and anxiety were initially identified using the MEDLINE-PubMed and Google Scholar databases using the terms “anxiety,” “OCD,” “GAD,” “obsessive-compulsive,” “generalized anxiety,” “worry,” “action monitoring,” “performance monitoring,” “conflict monitoring,” “error-related negativity,” “Ne,” and “ERN.” Additional studies were identified from the reference sections of the articles obtained from the online searches and from contacting investigators for additional unpublished datasets. This initial search yielded a total of 75 studies and datasets.

### Inclusion/exclusion criteria

**Figure 2:**
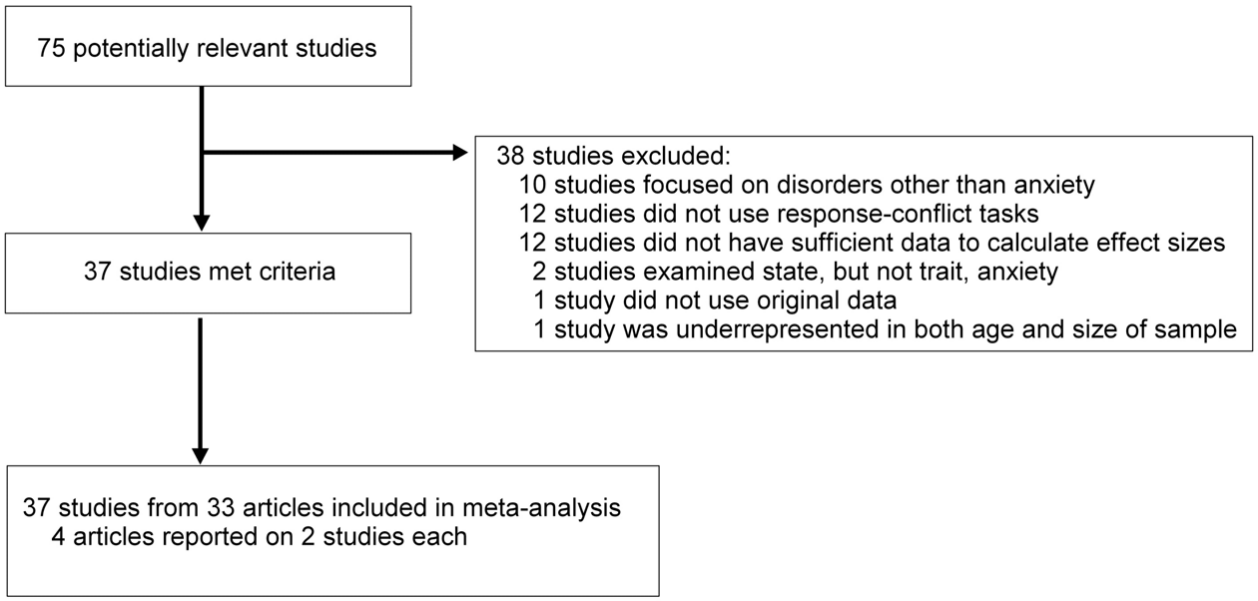
**Selection of studies**. Flow chart depicting the selection of studies used in the meta-analysis.

Figure 2 depicts the study selection process used for the meta-analysis. Studies were included in the current meta-analysis if ERN data were reported and they included a measure that specifically identified “anxiety” as the primary construct measured (e.g., the State-Trait Anxiety Inventory—Trait Version; STAI-T) or others tapping closely related constructs such as behavioral inhibition (Behavioral Inhibition System scale; BIS). We did, however, exclude studies in which anxiety was examined as secondary to a different primary psychopathology (e.g., secondary anxiety to a comorbid primary alcohol use disorder; Schellekens et al., 2010). Moreover, we focused on studies of the response-locked ERN elicited in standard conflict tasks, such as the Eriksen flanker task (Eriksen and Eriksen, 1974), the Stroop task (Stroop, 1935), or variants of the Go/No-Go task. Beyond our motivations described above, this decision is further justified by studies showing that enhanced ERN is uniquely associated with OCD diagnosis and symptoms in such response conflict tasks (Nieuwenhuis et al., 2005; Gründler et al., 2009; Mathews et al., 2012). We excluded studies using trial-by-trial motivation manipulations. Studies were also excluded if we were unable to compute a quantitative estimate (i.e., effect size) of the relationship between anxiety and the ERN. One study (Cavanagh et al., 2010) was excluded because it reported a re-analysis of data that were included in the final meta-analysis (Gründler et al., 2009; Study 2 Flankers task). Because Moser et al. (2012) reported on a subset of the full sample reported on in Moran et al. (2012) we only included the Moran et al. (2012) study so as to include the full sample. Moreover, we did not include the anxious arousal data from Moran et al. (2012) in the overall analysis, as the sample is entirely redundant with the anxious apprehension data, but we did include it in moderation analyses described below.

Using our inclusion/exclusion criteria, a total of 37 studies were included in the present meta-analysis (see Table 1). The selection of studies was nearly equally distributed among healthy adult volunteer samples (19; 51%) and anxiety-disordered samples (16; 43%), with the remaining two studies using samples with healthy children. Of the 37 studies, 27 (73%) used a version of the Eriksen flanker task, 5 (14%) used a Go/NoGo task, 4 (11%) used the Color Stroop task, and 1 (2%) used the Simon task. There were a number of different self-report (and parent-report) measures of anxiety used in the final selection.

**Table 1:** **Characteristics of studies included in the meta-analysis**.

**Study**	**Population**	**Task**	**Anxiety measure**	**Type**
Aarts and Pourtois, 2010^a^^,^^b^^,^^c^	Volunteers	Go/NoGo	STAI-T	M
Amodio et al., 2008^a^	Volunteers	Go/NoGo	BIS	AA
Beste et al., 2013^a^	Volunteers	Go/NoGo flanker	ASI	M
Boksem et al., 2006^a^	Volunteers	Letter flanker	BIS	AA
Carrasco et al., 2013^a^^,^^b^^,^^c^	Pediatric OCD	Arrow flanker	K-SADS-PL	AA
Carrasco et al., 2013^a^^,^^b^^,^^c^	Pediatric OCD	Arrow flanker	K-SADS-PL	AA
Carrasco et al., 2013^a^^,^^b^^,^^c^	Pediatric anxiety	Arrow flanker	K-SADS-PL	AA
Cavanagh and Allen, 2008^a^	Volunteers	Letter flanker	BIS	AA
Chang et al., 2010^a^	Volunteers	Letter flanker	ASR	M
Gehring et al., 2000^a^^,^^c^	OCD	Color stroop	SCID	AA
Gründler et al., 2009^c^	Volunteers	Letter flanker	OCI-R	AA
Hajcak et al., 2008^c^	Pediatric OCD	Simon	Y-BOCS	AA
Hanna et al., 2012^a^^,^^b^^,^^c^	Pediatric OCD	Arrow flanker	K-SADS-PL	AA
Inzlicht et al., 2009 study 1^a^	Volunteers	Color stroop	BIS	AA
Inzlicht et al., 2009 study 2^a^	Volunteers	Color stroop	BFI-N	M
Johannes et al., 2001^a^	OCD	Go/NoGo	SCID	AA
Kaczkurkin, 2013^a^^,^^b^^,^^c^	Volunteers	Letter flanker	OCI-R	AA
Ladouceuer et al., 2006^c^	Pediatric anxiety	Arrow flanker	K-SADS-PL	M
Larson and Clayson, 2011^a^^,^^b^^,^^c^	Volunteers	Arrow flanker	STAI-T	M
Larson et al., 2010^a^^,^^b^^,^^c^	Volunteers	Color stroop	STAI-T	M
Larson et al., 2011^a^^,^^b^^,^^c^	Volunteers	Arrow flanker	STAI-T	M
Luu et al., 2000^a^^,^^c^	Volunteers	Letter flanker	PANAS	M
Meyer et al., 2012^a^^,^^b^^,^^c^	Pediatric anxiety	Arrow flanker	Parent-SCARED	M
Moran et al., 2012^a^^,^^b^^,^^c^	Volunteers	Letter flanker	PSWQ	AA
Moran et al., 2012^a^^,^^b^^,^^c^	Volunteers	Letter flanker	MASQ-AA	M
Olvet and Hajcak, 2009^a^^,^^b^^,^^c^	Volunteers	Letter flanker	DASS	M
Olvet and Hajcak, 2012^a^^,^^b^^,^^c^	Volunteers	Arrow flanker	BFI-N	M
Rabinak et al., 2013^a^^,^^b^^,^^c^	Veterans	Arrow flanker	SCID	M
Riesel et al., 2011^a^^,^^b^	OCD	Arrow flanker	SCID	AA
Ruchsow et al., 2005^c^	OCD	Go/NoGo flanker	SCID	AA
Santesso et al., 2006^a^	Pediatric OC	Letter flanker	CBCL-OC	AA
Stern et al., 2010^a^^,^^b^^,^^c^	OCD	Letter flanker	SCID	AA
Tops and Boksem, 2011^a^^,^^b^^,^^c^	Volunteers	Letter flanker	BIS	AA
Weinberg et al., 2010^a^^,^^b^^,^^c^	GAD	Arrow flanker	SCID	AA
Weinberg et al., 2012a^a^^,^^b^^,^^c^	GAD	Arrow flanker	SCID	AA
Xiao et al., 2011^a^^,^^b^^,^^c^	GAD	Letter flanker	Chinese MINI	AA
Xiao et al., 2011^a^^,^^b^^,^^c^	OCD	Letter flanker	Chinese MINI	AA

a *ERN data available*.

b *CRN data available*.

c Δ*ERN data available*.

*Population Acronyms: GAD, Generalized Anxiety Disorder Patients; OCD, Obsessive-Compulsive Disordered Patients; OC, Obsessive-Compulsive*.

*Anxiety Measure Acronyms: ASI, Anxiety Sensitivity Index; ASR, Achenbach Self-Report; BFI-N, Big Five Inventory -Neuroticism; BIS, Behavioral Inhibition System Scale; CBCL, Child Behavior Checklist (OC, Obsessive-Compulsive Scale); DASS, Depression Anxiety Stress Scale; K-SADS-PL, Schedule for Affective Disorders and Schizophrenia for School-Age Children-Present and Lifetime Version; MASQ-AA, Mood and Anxiety Symptom Questionnaire: Anxious Arousal Subscale; MINI, Mini-International Neuropsychiatric Interview; OCI-R, Obsessive-Compulsive Inventory-Revised; PANAS, Positive and Negative Affect Schedule; PSWQ, Penn State Worry Questionnaire; SCARED, Screen for Child Anxiety Related Disorders; SCID, Structured Clinical Interview for DSM Disorders; STAI-T, State and Trait Anxiety Inventory-Trait Version; Y-BOCS, Yale-Brown Obsessive-Compulsive Scale*.

*Type refers to Anxiety Type; AA, Anxious Apprehension (worry); M, Mixed anxiety*.

*Nine (24%) of the studies included in the current meta-analysis were also reported on in the Mathews et al. (2012) meta-analysis*.

### Overview of analyses

For the present analysis, we used the varying-coefficient model^2^ recommended by Bonett (2008, 2009, 2010) and Krizan (2010) because (1) it does not rely on the unrealistic assumptions made by other fixed effects meta-analytic models (e.g., the existence of a single population effect size), (2) Bonett (2008, 2009, 2010) has demonstrated that varying-coefficient models provide more precise confidence intervals than other models, and (3) it performs well in the presence of correlation heterogeneity and non-randomly selected studies (Bonett, 2008; c.f. Brannick et al., 2011). Synthesizer 1.0 (Krizan, 2010) was used for computing point estimates and 95% confidence intervals (95% CIs).

Pearson's *r* was the focal effect size for all studies rather than Cohen's *d* as the former is more consistent with the idea that anxiety is a continuous dimension rather than a distinct category (Watson, 2005; Brown and Barlow, 2009). Cohen (1988) suggested that *r*s ranging between |0.1| and |0.29| represent small effects, *r*s ranging between |0.30| and |0.49| represent medium effects and *rs* exceeding |0.50| are considered large effects. When interpreting the results of the present analyses, it is useful to recall that error-monitoring ERPs are negative deflections—that is, a larger ERN is one that is more negative. Negative correlations therefore indicate that greater anxiety scores are associated with a more negative deflection whereas a positive correlation would indicate that anxiety is associated with a less negative deflection (i.e., a smaller ERN).

We attempted to obtain data for all measures from all published studies and known unpublished datasets, but complete coverage was not possible in all cases. Thus, many of the following analyses were conducted with subsets of the total number of datasets.

The first set of analyses aimed to quantify the overall relationships between anxiety—broadly defined—and ERN, CRN, and ΔERN. Effect sizes were computed across studies using the reported associations between anxiety measures or groups and the ERN. Most studies reported on a single anxiety-related measure or group. In some other cases, investigators included more than one anxiety-related measure. In these cases, we chose the anxiety-related measure that was most consistently used across studies so as to maximize the potential for comparability across studies.

The focal analyses tested the hypothesis that anxious apprehension is the dimension of anxiety most closely associated with the ERN (as well as the CRN and ΔERN). To do this, we created two groups of studies based on their measures of anxiety. The first group was called the “anxious apprehension” group, which included studies of GAD and OCD diagnoses and symptoms as well as studies of the BIS. Our decision to include the BIS in the anxious apprehension group was based on four considerations: (1) three of the seven items (42%) making up the BIS measure used in ERN research include the word “worry” (Carver and White, 1994); (2) a recent large-scale study demonstrated that anxious apprehension (as measured by the Penn State Worry Questionnaire; PSWQ) was nearly twice as highly correlated with an avoidance motivation factor, including a measure of BIS, than anxious arousal (as measured by the Mood and Anxiety Symptom Questionnaire—Anxious Arousal subscale; MASQ-AA; Spielberg et al., 2011); (3) data from our own research team indicates that anxious apprehension correlates three times as highly with BIS, itself, than anxious arousal^3^ and (4) existing theory that links BIS to anxious apprehension and conflict between competing responses (Gray and McNaughton, 2000; Barlow, 2002; Amodio et al., 2008). The second group of studies was called the “mixed” group, which included all other studies. Our reasoning for grouping all other studies together was that they involved non-specific measures of anxiety-related constructs that often mix anxious apprehension with anxious arousal (e.g., the Anxiety Sensitivity Index; ASI) or combine anxiety with depression-related symptoms (e.g., STAI-T). To formally test our differential specificity hypothesis, we compared the magnitude of the aggregated correlation coefficients between the anxious apprehension and mixed studies using Synthesizer software (Krizan, 2010).

## Results and interim discussion

**Figure 3:**
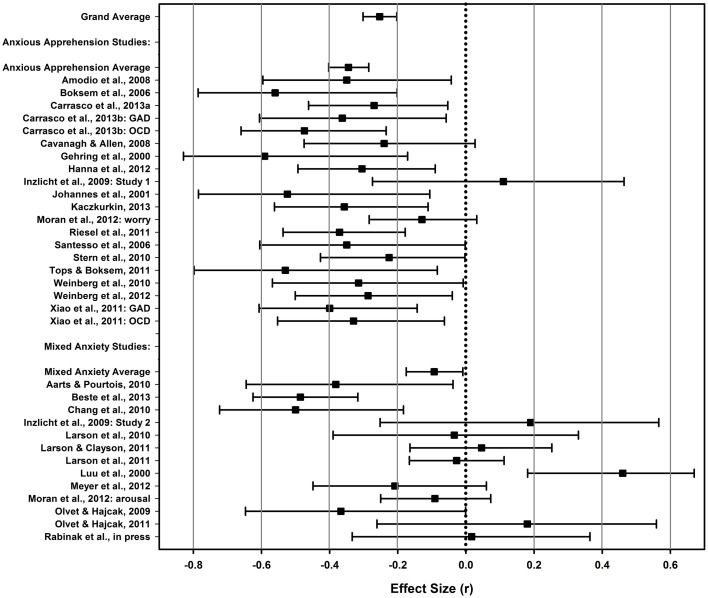
**ERN forest**. A forest plot depicting effect sizes (*r*) between the ERN and measures of anxiety for the meta-analytic average (top), the anxious apprehension and mixed anxiety averages, and individual studies. Error bars depict the 95% confidence interval for the effect size. The dotted line indicates an effect size of 0.

**Figure 4:**
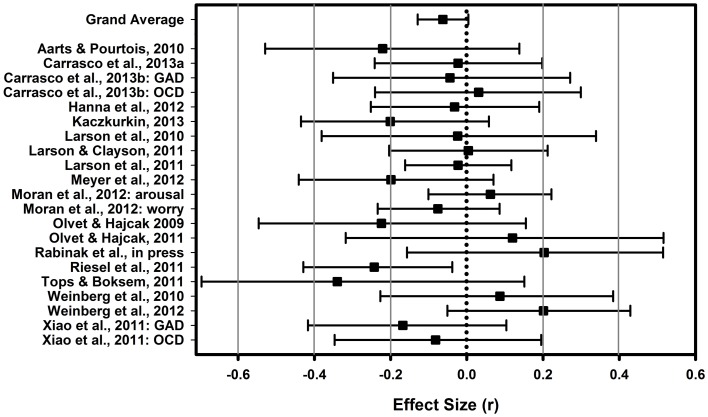
**CRN forest**. A forest plot depicting effect sizes (*r*) between the CRN and measures of anxiety for the meta-analytic average (top) and individual studies. Error bars depict the 95% confidence interval for the effect size. The dotted line indicates an effect size of 0.

**Figure 5:**
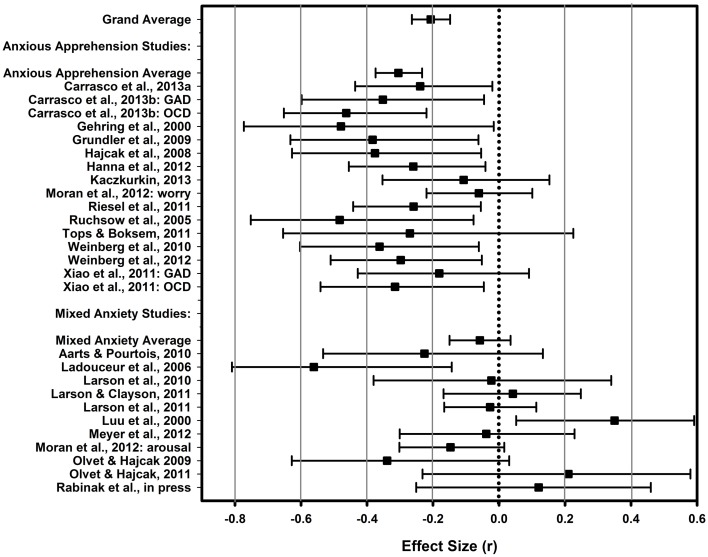
**ΔERN forest**. A forest plot depicting effect sizes (*r*) between the ΔERN and measures of anxiety for the meta-analytic average (top), the anxious apprehension and mixed anxiety averages, and individual studies. Error bars depict the 95% confidence interval for the effect size. The dotted line indicates an effect size of 0.

See Table 2 for details of the results. Overall, we found that anxiety—broadly defined—demonstrated a small to medium association with the ERN and ΔERN. The CRN, however, was not reliably associated with anxiety symptoms. Critical to our focal hypothesis, we confirmed that anxious apprehension was more strongly related to enhanced ERN than non-specific, “mixed,” forms of anxiety-related symptoms (see Table 2). The relationships between anxious apprehension and the ERN and ΔERN were medium in size whereas the relationships between mixed anxiety and the ERN and ΔERN were quite small (*r*s < 0.10). Results from individual studies for the ERN, CRN, and ΔERN can be found in Figures 3–5, respectively. As can be gleaned from the figures, the mixed anxiety studies were much more variable in their effect sizes, with many studies showing very large confidence intervals as well. Estimates of the CRN effect sizes were likewise quite variable and, in all but one study, demonstrated non-significant results. Together, these results support the notion that the association between error-related brain activity and anxious apprehension is robust whereas the association with less specific forms of anxiety is significantly weaker. Moreover, given the non-specific nature of the measures employed in the “mixed” studies, it is also possible that any associations we detected may, in fact, be driven by the anxious apprehension-related items.

**Table 2:** **Results from the meta analysis**.

**Sample**	***r***	***n***	***k***	**95% CIs**	***r*_diff_**	**95% CIs_−diff_**
**ERN**						
Overall^†^	−**0.253**	1757	32	−**0.302**; −**0.203**	**0.253**	**0.153**; **0.370**
Apprehension	−**0.345**	1077	20	−**0.403**; −**0.285**	–	–
Mixed	−**0.093**	826	13	−**0.175**; −**0.009**	–	–
**CRN^a^**						
Overall	−0.063	1264	20	−0.129; 0.004	0.041	−0.086; 0.168
**ΔERN**						
Overall	−**0.207**	1437	26	−**0.264**; −**0.148**	**0.247**	**0.132**; **0.375**
Apprehension	−**0.305**	889	16	−**0.374**; −**0.233**	–	–
Mixed	−0.058	694	11	−0.150; 0.035	–	–

a *Only one effect is presented for the CRN as no moderation was found (see Table 2)*.

Key:

*r: aggregate effect size of association with anxiety*.

*n: is the total number of participants across all studies*.

*k: number of studies/samples*.

*95% CIs: 95% confidence intervals for the aggregate correlation (bold type indicates that the confidence interval does not include 0)*.

**r*_diff_: difference between the aggregate effect sizes between anxious apprehension and mixed anxiety*.

*95% CIs_-diff_: 95% confidence intervals for the difference (bold type indicates that the confidence interval does not include 0). Adjusting for three comparisons, these moderator analyses remain significant*.

† *In the initial analysis, we did not include the anxious arousal data from Moran et al. (2012) as the sample is entirely redundant with the anxious apprehension data. When the anxious arousal data from are included, the ERN (r = −0.25, k = 33, n = 1903, 95% CIs: −0.30; −0.20) and ΔERN (r = −0.21, k = 27, n = 1583, 95% CIs: 0.26; −0.15) continued to show significant associations with anxiety*.

One concern is that nearly all studies conducted with patient samples were included in the anxious apprehension group thus potentially conflating the dimension of anxiety under study with patient status^4^. To address this issue, we tested moderation for the ERN using non-patient studies; the mixed anxiety group contained only a single patient study thus precluding our ability to test moderation for the patient studies. After removing patient studies, anxious apprehension studies (*r* = −0.301, *k* = 8; *n* = 410; 95% CIs: −0.400; −0.195) continued to show greater effect sizes than mixed anxiety studies (*r* = −0.101, *k* = 12, *n* = 794; 95% CIs: −0.186; −0.016; *r*_*diff*_ = 0.199; 95% CIs for the difference: 0.064; 0.349). Therefore, the difference in effect sizes between the anxious apprehension vs. mixed anxiety studies cannot be accounted for by patient studies alone.

All told, the results of the current meta-analysis indicate that anxiety, broadly defined, demonstrates a small to medium association with ERP indices of error monitoring. Most importantly, the findings are consistent with the hypothesis that an enhanced ERN is more strongly associated with the anxious apprehension dimension of anxiety as opposed to other anxiety-related constructs. Specifically, associations between anxious apprehension and ERN and ΔERN were more than three times as large as those with other forms of anxiety^5^. In contrast, anxiety showed no reliable association with the CRN, irrespective of the way in which anxiety was operationalized. This finding provides critical information for developing mechanistic models of the links between anxiety and error monitoring. Before detailing a conceptual framework to understand these findings, however, it is useful to point out caveats regarding the current meta-analysis and present a few practical considerations for future research.

First, the current meta-analysis included a relatively small number of studies. However, this is the first meta-analysis of its kind and the total number of studies (*N* = 37) is in line with previous meta-analyses of associations between psychopathology and ERPs (e.g., Polich et al., 1994; Bramon et al., 2004; Mathews et al., 2012). Second, the precision of effect size estimates will also be improved if researchers collect larger samples than have typically been used in this literature to date (sample sizes in the current analysis were as low as *n* = 18; Median = 40, *SD* = 40.49), especially because most effect sizes in the social sciences are relatively small (Cohen, 1988; Richard et al., 2003).

Third and most importantly, the task of pin-pointing the association between type of anxiety and error monitoring has received limited attention in the literature. Most studies have taken a more global approach by focusing on individuals with symptoms of GAD or OCD, or by considering associations between relatively generic anxiety symptoms and error monitoring ERPs. We are aware of only two studies that have attempted to empirically isolate specific relationships between facets of anxiety and error monitoring: our recent study (Moser et al., 2012) showing that anxious apprehension was more related to enhanced ERN than anxious arousal and Hajcak and colleagues' (2003) study showing that high anxious apprehensive students showed enhanced ERN compared to spider phobic students. With the current meta-analysis we aimed to significantly extend this line of research. However, because so little data exist that parse dimensions of anxiety in relation to the ERN, we had to create groups of studies, many of which included overall measures that tap a variety of anxiety-related constructs.

We acknowledge that we took a conservative approach to classifying the content of specific measures and compared studies that used fairly clear measures of anxious apprehension—GAD and OCD-related measures—to all others. It is evident from the effect size estimates and figures that there is much more consistency of positive findings in the studies using more precise measures of anxious apprehension. Ideally, there would be more studies directly comparing ERN magnitudes across groups of participants created using targeted instruments of different anxiety constructs—e.g., anxious apprehension vs. anxious arousal. This is a challenge we hope future research will undertake, as it is not only important to the current topic but also to building a more biologically informed rubric for mental disorder classification (cf. Cuthbert and Insel, 2010; Sanislow et al., 2010). In this way, our current analyses build on seminal work by Heller and colleagues that has differentiated anxious apprehension from anxious arousal across psychometric and physiologic studies (Heller et al., 1997; Nitschke et al., 1999, 2001; Engels et al., 2007; Silton et al., 2011; Spielberg et al., 2011).

In the next section, we use the results of this meta-analysis as a starting point for building a conceptual framework to explain why anxious apprehension/worry is the dimension of anxiety most closely associated with enhanced ERN. In short, we propose a *compensatory error-monitoring hypothesis* to explain the association between anxiety and enhanced ERN. Our core claim is that enhanced ERN in anxiety results from the interplay of a decrease in processes supporting active goal maintenance, because of the distracting effects of worry, and a compensatory increase in processes (e.g., effort) dedicated to transient reactivation of task goals on an as-needed basis when errors occur.

## Discussion

### The compensatory error monitoring hypothesis

Our conceptual framework is an extension of existing affective-motivational models of the association between anxiety-related constructs and enhanced ERN (Luu and Tucker, 2004; Pailing and Segalowitz, 2004; Weinberg et al., 2012a,b). The foundation of our account rests on four key findings about anxiety and cognitive function: (1) that anxious apprehension/worry is significantly involved in cognitive abnormalities in anxiety, (2) that anxious performance is characterized by processing inefficiency, (3) that enhanced ERN in anxiety is observed without corresponding deficits in task performance, and (4) that individuals with anxiety exhibit enhanced transient “reactive” control but reduced preparatory “proactive” control. We further incorporate the conflict monitoring theory of the ERN (Yeung et al., 2004) in order to cast the anxiety-ERN relationship in more mechanistic terms.

#### The role of anxious apprehension/worry

The present proposal builds on our earlier explanation for why anxious apprehension shows a particularly strong association with enhanced ERN (Moran et al., 2012; Moser et al., 2012), which in turn drew heavily on Eysenck and colleagues' (2007) Attentional Control Theory (ACT). ACT is a recent extension of Eysenck and Calvo's (1992) original Processing Efficiency Theory (PET), which itself drew on Sarason's (1988) earlier Cognitive Interference Theory. What all of these theories have in common is their emphasis on the deleterious effects of anxious apprehension on cognition. That is, all posit that distracting worries interfere with the ability of anxious individuals to stay focused on affectively neutral cognitive tasks. These early theories were supported by several studies showing the specific effects of worry on cognitive performance (e.g., Morris et al., 1981).

ACT increased specificity of the earlier work by proposing that anxiety is associated with a deficit in attentional control that results from an imbalance in activity between the frontal goal-directed attention system—concerned with goals and plans—and the parietal stimulus-driven attention system—concerned with salience and threat. Specifically, the ACT suggests that anxious individuals are characterized by enhanced activity of the stimulus-driven attention system and decreased functionality of the goal-driven system. Anxious individuals are therefore tuned to prioritize salient internal (e.g., worry) and external (e.g., angry face) sources of potential threat at the expense of affectively-neutral task-relevant stimuli. When no source of external threat or distraction is present (e.g., during performance of a standard conflict task) worry is distracting and likely to deplete goal-driven resources. Our initial formulation of the anxiety-ERN relationship (Moran et al., 2012; Moser et al., 2012) applied this common assertion that the worry component of anxiety is responsible for cognitive processing abnormalities in affectively-neutral tasks, using this idea to explain that this anxiety dimension, in particular, is most closely related to the ERN.

The notion that anxiety's influence on cognitive performance is primarily the result of the distracting effects of worry also appears as the cornerstone of work by Beilock and colleagues (Beilock and Carr, 2005; Beilock, 2008) who study relationships between anxiety and academic performance. Beilock (2008, 2010) suggests that worry co-opts available working memory resources that would otherwise be allocated to the task at hand. Their work has demonstrated that a variety of types of academic anxiety—from math anxiety to spatial anxiety (Ramirez et al., 2012)—impair performance because of worry's drain on resources. Thus, there is significant precedent from a variety of researchers for focusing on the unique effects of worry on cognition in anxiety.

#### Anxiety is associated with processing inefficiency

As initially noted by Eysenck and Calvo (1992) in their seminal review paper on Processing Efficiency Theory, anxious individuals often perform just as well as their non-anxious counterparts. The reason performance is spared, they suggested, is that anxious individuals employ compensatory effort because, although worries are distracting, they also motivate anxious individuals to overcome the negative effects of their anxiety on performance. This dual-pathway compensatory effort idea helped to reconcile inconsistencies in the literature regarding the effects of anxiety on performance.

How did they come to hypothesize the role of compensatory effort? First, Eysenck and colleagues showed that anxiety is often related to longer reaction times, but intact accuracy, across a range of reasoning, reading, attention, and working memory tasks (as reviewed by Eysenck and Calvo, 1992 and later again by Eysenck et al., 2007). Thus, to achieve the same level of performance accuracy seems to require anxious individuals to deploy enhanced effort and processing resources that take longer to implement. Second, their reviews showed that anxious individuals also self-report using more effort on tasks in which they perform at the same level as non-anxious individuals. PET and ACT therefore suggest that anxiety is associated with *processing inefficiency*—more effort or resources allocated to achieve comparable level of accuracy—but not necessarily ineffectiveness (i.e., low accuracy).

More recently, neuroimaging studies have provided additional support for the claim that enhanced processing resources (compensatory effort) help anxious individuals maintain typical levels of performance (for a review see Berggren and Derakshan, 2013). For example, enhanced dorsolateral prefrontal cortex activity was reported on incongruent relative to congruent Stroop trials in a sample of anxious college students (Basten et al., 2011). Similarly, enhanced NoGo N2 was reported in anxious students despite comparable performance to non-anxious students (Righi et al., 2009). Berggren and Derakshan (2013) summarized a number of additional consonant effects—i.e., greater processing resources and compensatory effort revealed in anxious individuals despite comparable behavioral performance –across a range of attention and memory paradigms.

In addition, a recent neuroimaging study showed that anxiety's deleterious effect on math performance was curtailed to the extent that high math anxious participants recruited frontal control brain regions (Lyons and Beilock, 2011). Thus, the impact of anxiety on academic performance was mitigated by compensatory cognitive control—precisely as PET/ACT would predict. There is therefore strong support for the notion that anxious individuals can perform as well as non-anxious individuals; however, they draw on more processing resources and effort to do so.

Directly related to the ERN, processing inefficiency provides an explanation for a curious finding from Endrass et al. (2010) who showed that although non-anxious control participants demonstrated an enhanced ERN during a punishment condition, OCD patients did not. Specifically, ACT (Eysenck et al., 2007) predicts that motivational manipulations should have minimal impact on anxious individuals because compensatory effort is already being employed during baseline performance whereas such manipulations should cause increases in performance in non-anxious individuals because they allocate more effort to achieve the incentive. Indeed, Eysenck and colleagues demonstrated this effect in early behavioral work (as reviewed in Eysenck et al., 2007). In this light, Endrass and colleagues' (2010) results suggest that enhanced ERN in non-anxious individuals during punishment reflected increased compensatory error monitoring that was already at ceiling in the OCD group during the standard condition.

#### Enhanced ERN in anxiety is observed in the absence of compromised performance

Consistent with PET/ACT and the above-reviewed studies, anxious individuals seem to demonstrate typical levels of performance in the standard conflict tasks used in ERN studies. Yet, they consistently show enhanced ERN. Indeed, only three individual studies of the 37 included in the present meta-analysis of the ERN reported a significant relationship between anxiety and error rate. A binomial test suggests that this is consistent with a 5% false positive rate (*z* = 1.02, *p* = 0.16). Moreover, no individual study reported a significant relationship between anxiety and reaction time.

To further evaluate this issue, we conducted an additional meta-analysis on error rate and reaction time for those studies reported on in our meta-analysis of the ERN. As we did with the ERN, we first conducted the meta-analysis across all studies for which we could calculate effect sizes. Then, we conducted moderation analysis by anxiety type. This analysis yielded no significant relationship between anxiety (across all studies) and error rate (*k* = 29; *N* = 1668; *r* = −0.02, 95% CIs: −0.08; 0.03). There was, however, significant moderation by anxiety type such that anxious apprehension was associated with lower error rate (*r* = −0.08; 95% CIs: −0.15; −0.004) and mixed anxiety was associated with non-significantly higher error rate (*r* = 0.08; 95% CIs: −0.02; 0.18; *r*_*diff*_ = 0.16, 95% CIs for the difference: 0.04; 0.28). Both of these effects are notably small in magnitude. With regard to overall reaction time, there was no significant effect of anxiety (*k* = 26; *N* = 1480; *r* = −0.06, 95% CIs: −0.12; 0.002), nor was there any significant evidence of moderation (*r*_*diff*_ = 0.09; 95% CIs: −0.05; 0.23). Together, these findings suggest the small-to-medium association between anxiety (across all studies) and the ERN is observed in the absence of altered behavioral performance. Importantly, the associations between error rate and anxious apprehension and mixed anxiety unlikely contribute to ERN effects, as they emerge as small effects and in opposing directions for the two anxiety types.

Thus, in line with the notion that anxiety is characterized by processing inefficiency, we suggest that enhanced ERN in anxiety may index a compensatory effort signal aimed at maintaining a standard level of performance (Moran et al., 2012; Moser et al., 2012). That is, enhanced ERN related to anxiety reflects *inefficient error monitoring*, in that anxious individuals may rely on greater error monitoring resources to achieve the same level of performance as non-anxious individuals. Together, then, we suggest that the specific distracting effects of worry during affectively-neutral conflict tasks requires anxious individuals to engage in compensatory effort to perform up to par, with enhanced ERN being one index of this compensatory effort/greater utilization of processing resources.

#### Anxiety is associated with enhanced reactive control, but reduced proactive control

Braver (2012) and colleagues' (Braver et al., 2007) dual mechanisms of control (DMC) model provides another compatible context in which to understand the role of enhanced ERN as a compensatory effort signal in anxiety. The DMC model suggests that cognitive control is achieved through two distinct modes: proactive and reactive. Proactive control—the more cognitively taxing of the two modes—involves active maintenance of rules and goals within lateral areas of prefrontal cortex in a *preemptive* fashion to facilitate future performance. In contrast, reactive control—the less effortful mode—involves allocating attention to rules and goals on an *as-needed* basis, once a problem (such as the occurrence of conflict or an error) has arisen. Furthermore, Braver (2012) refers to reactive control as a “‘late correction' mechanism” (p. 106) and links it to activity of the ACC, such that ACC-mediated conflict monitoring may help individuals reactivate task goals in a transient, as-needed fashion. The DMC model is therefore immediately relevant to the current discussion because it directly parallels the focus of ACT on the interaction between goal-driven (or proactive control) and stimulus-driven (or reactive control) attention systems (Eysenck et al., 2007).

According to Braver (2012), non-anxious individuals are able to alternate flexibly between reactive and proactive control modes in accordance with changing task demands. In contrast, Braver (2012) suggests that anxious individuals are distracted by worries that deplete resources needed for active goal maintenance, thereby interfering with proactive control and throwing chronically anxious individuals into a reactive control mode. That is, anxious individuals rely more heavily on reactive control. Increasing evidence supports this propensity for anxious individuals to preferentially engage in reactive control (Gray et al., 2005; Fales et al., 2008; Krug and Carter, 2010, 2012). For example, Fales et al. (2008) showed that anxious individuals demonstrated decreased sustained, but increased transient, activity in working memory regions consistent with the notion of decreased proactive and increased reactive control.

A recent study by Nash et al. (2012) showing that increased behavioral activation system (BAS) activity, as indexed by left-sided prefrontal EEG asymmetry, was associated with a reduced ERN provides additional support for our proposed differential effects of proactive and reactive control on ERN. Indeed, BAS has been associated with proactive control and reduced dACC/MCC activity (see Braver et al., 2007 for a review). Thus, while anxiety/BIS is associated with reactive control and therefore an enhanced ERN—as demonstrated in our meta-analysis—BAS is associated with proactive control and therefore a reduced ERN.

#### Formalizing the model using the conflict monitoring theory of the ERN

We adopt the conflict monitoring theory of the ERN and its recent extensions (Yeung and Cohen, 2006; Steinhauser and Yeung, 2010; Hughes and Yeung, 2011; Yeung and Summerfield, 2012) so as to leverage a well-articulated computational model of the ERN to help explain the relationship between anxiety and enhanced ERN. According to the conflict monitoring theory, the ERN reflects conflict that is detected when continued target processing after an error leads to activation of the correct response, resulting in conflict with the error just produced. This notion is rooted in the classic finding that individuals tend to automatically correct their mistakes as a result of continued stimulus processing (Rabbitt, 1966; Rabbitt and Vyas, 1981). Thus, the ERN indexes processes involved in the rapid correction of errors that reflects the current level of cognitive demand or effort—i.e., the level of response conflict (see also Hughes and Yeung, 2011; Yeung and Summerfield, 2012). In the context of broader theories of the ACC—the neural source of the ERN—the ERN provides information about current conflicts in order to optimize action selection and behavior (Botvinick et al., 2001; Botvinick, 2007). The conflict monitoring theory of the ERN nicely dovetails with the DMC in that both suggest the ACC is involved in reactive control, insofar as the ERN reflects ACC-mediated conflict monitoring arising from activation of the error-correcting response (Yeung and Summerfield, 2012)—i.e., a late correction mechanism.

Thus, our *compensatory error-monitoring hypothesis* of enhanced ERN in anxiety first draws on the above reviewed theory and evidence in assuming that anxiety increases sustained attention to internal sources of threat (i.e., worry) thereby reducing available resources dedicated to active maintenance of task rules and goals. As a result, the anxious individual is forced to rely on reactive control as a compensatory strategy. *Critically, when errors occur, reactive control causes an increase in stimulus processing around and after the time of the incorrect response, leading to enhanced conflict between the just-produced error and the correct (target) response that gives rise to an enhanced ERN (Yeung et al., 2004). Detection of this conflict could then help to reactivate task goals in the moment and normalize performance in anxious individuals (Braver, 2012)*. At least with respect to conflict tasks, this dynamic seems to provide a mechanistic account of an enhanced ERN in the presence of comparable performance among anxious individuals, because the interactive effects of reduced proactive control and increased reactive control would cancel each other out at the behavioral level. Having detailed our compensatory error-monitoring hypothesis, we now turn to new sources of evidence that provide additional support for our claims.

#### New sources of support for the compensatory error-monitoring hypothesis

Our compensatory error-monitoring hypothesis largely hinges on two ideas: (1) that the cognitive load of worry begins a cascade of processes that lead to enhanced ERN in anxious individuals, and (2) enhanced ERN in anxiety reflects a compensatory attention/effort response. In this section, we present data from our own lab that provides more direct support for these underlying assertions of our model.

If enhanced ERN in anxiety results from the cognitive load of worries on processing resources, it follows that experimentally induced cognitive load should also lead to enhanced ERN. Recent experimental data from our lab supports this notion that cognitive load—an affectively-neutral analog to distracting worries—enhances the ERN. In a study by Schroder et al. (2012), we showed that the ERN is enhanced when stimulus-response rules are switched, resulting in the need for individuals to simultaneously inhibit old rules and maintain current rules. We suggested that as a result of this need to juggle old and current rules, a cognitive load was placed on subjects during trials in which stimulus-response rules were switched. When errors occurred, then, compensatory attentional effort was employed as a reactive control strategy resulting in enhanced ERN.

More directly, we conducted an experiment examining the effect of verbal working memory load (WML) on the ERN (Moran and Moser, 2012), the details of which we present here. Twenty-nine undergraduates (21 Female, *M* age = 19.52 years, *SD* = 2.72) completed a flanker task interleaved with a successor-naming task (for a similar method, see (Lavie and Defockert, 2005): Experiment 2). Prior to each flanker stimulus, participants saw a string of five numbers to remember. Each five-number string was either in numerical order (low WML) or in a random order (high WML). Participants were instructed to memorize these digits. Following each flanker stimulus, a memory probe, which consisted of a randomly-selected number from the five-number memory set, was presented and participants were instructed to input the digit that followed the memory probe digit in the memory set for that trial. The experimental session consisted of 480 trials grouped into six blocks. Load was randomly varied by block such that a given block contained only one type of WML. There were an equal number of high- and low-WML blocks. The ERN (and CRN) elicited by flanker errors was calculated as the average activity in the 0–100 ms post-response time window relative to a −200 to 0 ms pre-response baseline at FCz. ERN/CRNs were then submitted to a 2 (Accuracy: Error vs. Correct) × 2 (WML: High vs. Low) repeated-measures analysis of variance (ANOVA).

**Figure 6:**
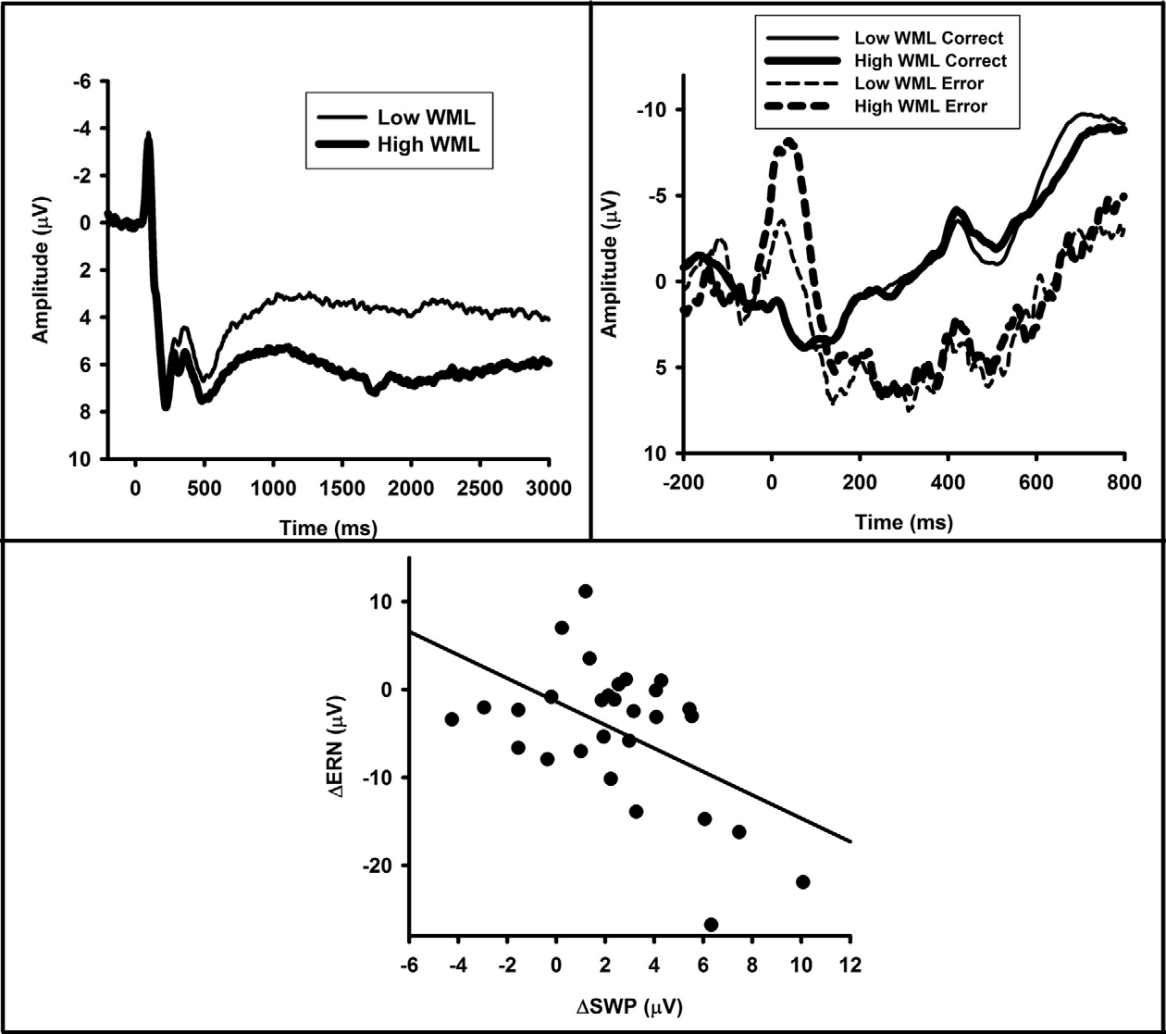
**Working memory load enhances ERN. (Top Left)** SWPs elicited during the memory retention interval. **(Top Right)** Response-locked ERPs as a function of accuracy and WML. **(Bottom)** A scatterplot depicting the association between WM-related changes in SWPs and ERNs.

Of key interest was the prediction that ERN amplitude should be increased in conditions of increased WM load. The main effect of accuracy [*F*_(1, 28)_ = 39.54, *p* < 0.01, η ^2^_*p*_ = 0.59] confirmed the presence of a clear ERN in this paradigm. Crucially, and consistent with our hypothesis, the WML × accuracy interaction was significant [*F*_(1, 28)_ = 9.69, *p* < 0.01, η ^2^_*p*_ = 0.28; See Figure 6 top right panel]. The ERN was enhanced on high load trials [*t*_(28)_ = 3.50, *p* < 0.01] whereas the CRN was unaffected by the WML manipulation (*t* < 1). Moreover, the ERN-CRN difference wave was greater on high WML trials than low WML trials [*t*_(28)_ = 3.11, *p* < 0.01].

To test the prediction that individual differences in sensitivity to load should correlate with changes in ERN amplitude, we also correlated the ERN with a well-validated ERP index of WM-retention. In particular, we measured the left-anterior positive slow-wave potential (SWP) that shows greater magnitude on high- vs. low-WML trials (Ruchkin et al., 1997; Berti et al., 2000; Kusak et al., 2000). By examining the relationship between the SWP (WM-retention) and the ERN, we intended to provide evidence that occupying WM functions under load, like worry, directly leads to increased ERN. The SWP was computed across the 500–3000 ms post-stimulus window with respect to a baseline consisting of the average activity in the 200 ms window immediately prior to the presentation of the memory set. The SWP was quantified as the average activity recorded at F3. SWPs were submitted to a single-factor (WML: High vs. Low) repeated-measures ANOVA.

Consistent with previous work, high WML memory sets elicited greater left-anterior positivity than low WML memory sets during the rehearsal period [*F*_(1, 28)_ = 18.21, *p* < 0.01, η ^2^_*p*_ = 0.39; see Figure 6 top left panel]. To directly link WM operations with the ERN, we first computed WM-related changes for each of our measures: ΔERN was computed as the ERN-CRN difference on high WML trials minus the ERN-CRN difference on low WML trials—that is, the extent to which error-related brain activity was modulated by the WML manipulation; ΔSWP was computed as the difference in activity between high and low WML trials during memory-set presentation. We focused on the ERN-CRN difference due to the significant Accuracy × WML interaction. However, if we compute ΔERN as the ERN on high-WML trials minus the ERN on low-WML trials the interpretation of the results does not change. Critically, findings revealed that ΔERN was strongly related to ΔSWP (*r* = −0.51, *p* < 0.01) indicating that enhanced ERN under high WML can be attributed to increased WM operations during rehearsal (Figure 6 bottom panel). Such data provide particularly strong causal evidence that current cognitive load leads to enhanced ERN. Together, they provide a proof-of-concept for the notion that the enhanced ERN that characterizes anxiety may result from WML imposed by worry.

Regarding our assertion that enhanced ERN in anxiety reflects a compensatory attention/effort response, we present results from a novel analysis examining associations between anxious apprehension, ERN, and academic performance—as measured by grade-point average (GPA)—on a subsample of data from a larger dataset (Moran et al., 2012). Past work has shown that larger ERN amplitudes correlate with higher GPA, suggesting that enhanced cognitive control is associated with higher academic achievement (Fisher et al., 2009; Hirsh and Inzlicht, 2010). However, no studies have examined whether anxiety moderates this relationship. We predicted that if enhanced ERN in anxious apprehension reflects a reactive compensatory control signal, a larger ERN in worriers should be associated with higher GPA. Following this logic, a low ERN in worriers would be associated with poorer academic performance. If, on the other hand, the ERN is not related to compensatory control in anxiety, the ERN-GPA relationship should not differ as a function of anxiety.

We tested these predictions in 59 undergraduates (24 female, *M* age = 20 years, *SD* = 3.20) who had useable cumulative GPA data collected from the University's Office of the Registrar. EEG recording procedures and task descriptions have been described elsewhere (Moran et al., 2012); participants engaged in a letter flanker task and then completed the Penn State Worry Questionnaire (PSWQ; Meyer et al., 1990). The ERN was calculated as the average activity in the 0–100 ms post-response time window relative to a −200 to 0 ms pre-response baseline correction at FCz (where it was maximal) on error trials.

**Figure 7:**
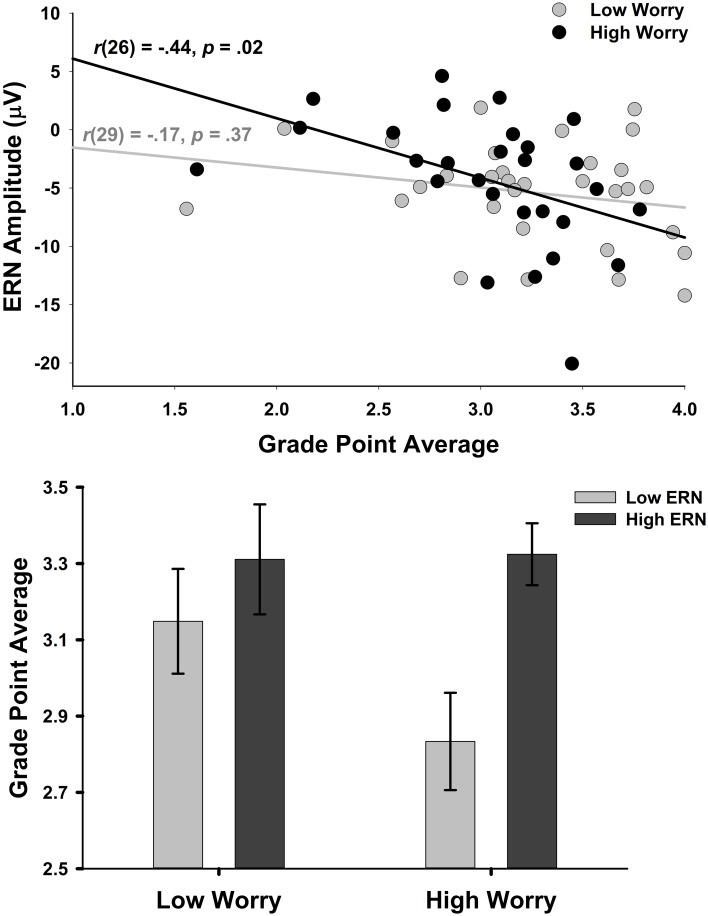
**Relationship between ERN and GPA is moderated by worry. (Top)** Scatterplot showing the relationship between ERN and GPA in the top 50% of the PSWQ distribution (black) and the bottom 50% (gray). **(Bottom)** Bar graph depicting GPA as a function of ERN and Worry groups which were created by median splits and described in the text. Error bars represent standard error of the mean.

Consistent with previous work (Hirsh and Inzlicht, 2010), larger ERN amplitude was significantly correlated with higher GPA across the whole sample (*r* = −0.30, *p* < 0.05). However, the relationship was small and non-significant among individuals below the median on PSWQ scores (Low Worriers, *n* = 31; *r* = −0.17, *p* = 0.37) but was significant and more than double the size among those above the median on the PSWQ (High Worriers, *n* = 28; *r* = −0.44, *p* < 0.05, see Figure 7). To explore further the relationships between worry, ERN amplitude, and GPA, the median scores on the PSWQ (Median = 51.00) and ERN (Median = −4.42 μV) were used to categorize participants into one of four groups: Low Worry—Low ERN (*n* = 13), High Worry—Low ERN (*n* = 16), Low Worry-High ERN (*n* = 18), and High Worry—High ERN (*n* = 12). A one-way analysis of variance (ANOVA) with Worry-ERN Group as the between-subjects factor and cumulative GPA as the dependent variable revealed a significant effect of Group [*F*_(3, 58)_ = 3.17, *p* = 0.03]. This effect is depicted in Figure 7. Fisher's least significant difference procedure indicated that participants in the High Worry-High ERN group had a significantly higher GPA (*M* = 3.32, *SD* = 0.53) than the High Worry-Low ERN group (*M* = 2.83, *SD* = 0.51; *p* < 0.05) and that the Low Worry-High ERN group (*M* = 3.31, *SD* = 0.61) also had a significantly higher GPA than the High Worry-Low ERN group (*p* < 0.01). The difference between the Low Worry-Low ERN group (*M* = 3.15, *SD* = 0.50) and High Worry-Low ERN group was marginal (*p* = 0.10). Critically, the High Worry-High ERN and Low Worry-High ERN groups did not differ on GPA (*p* > 0.90).

Together, these exploratory analyses provide further evidence that enhanced ERN among worriers functions as a compensatory control signal insomuch as worriers with a large ERN achieved the same GPA as non-worriers. In contrast, individuals with high worry and a low ERN, suggesting a lack of effortful compensatory control, tended to have significantly poorer academic achievement. Although preliminary, these findings are consistent with the Lyons and Beilock (2011) study showing that anxiety's deleterious effect on math performance was curtailed to the extent that high math anxious participants recruited frontal control brain regions.

### Predictions and directions for future research

To this point, we have provided theoretical rationale and empirical evidence for our *compensatory error-monitoring hypothesis* of the association between anxious apprehension and enhanced ERN. In this next section, we develop a set of additional predictions and key avenues for future research to pursue.

The first, and perhaps most obvious, prediction for future research to test is that inducing worry should lead to an enhancement of the ERN. Borkovec and Inz (1990) have developed and implemented a standard worry induction procedure for decades that could be easily utilized in the context of an ERN study. Previous anxiety inductions have demonstrated negative results with regard to their effects on the amplitude of the ERN. For instance, Moser et al. (2005) induced fear in spider phobic undergraduates and showed no effect on ERN magnitude. Similarly, Larson et al. (2013) failed to show an effect of an anxiety induction on ERN magnitude. Our prediction is that enhanced ERN will only be elicited to the extent that anxious apprehension—worry—is induced. The failure of existing studies to find effects of anxiety induction on ERN may therefore be the result of their use of anxious arousal inductions instead of worry inductions.

Similarly, we predict that worries captured at ERN testing should relate to enhanced ERN and may mediate the association between trait worry and enhanced ERN. Specifically, on- and/or off-task worries could be measured following flanker performance and related to the ERN. If worries during task performance are responsible for co-opting goal-driven resources and causing compensatory deployment of reactive control resources, then such measures of worry should relate to enhanced ERN. The Cognitive Interference Questionnaire (CIQ; Sarason and Stoops, 1978; Sarason et al., 1986) would be one measure of this construct worth exploring in this regard. Self report and thought sampling methods for measuring mind wandering and task-unrelated thoughts (Matthews et al., 1999; Schooler et al., 2011; Mrazek et al., 2011, 2013) would also be important for future tests of our hypotheses.

Following from our formulations and the preliminary findings of Endrass et al. (2010), we would also predict that incentive and motivation manipulations should have less effect on ERN amplitude in anxious than non-anxious populations. There are numerous ways to manipulate incentive and motivation and thus this effect could be tested in a variety of contexts. Previously, Hajcak et al. (2005) showed that the amplitude of the ERN was enhanced on trials that were worth more points toward a monetary incentive as well as under a condition of performance evaluation. We predict that such manipulations would not lead to enhanced ERN in anxious individuals because they already employ compensatory effort during baseline conditions.

Treatment studies not only offer the chance to help improve anxious peoples' functioning but also to test theory-derived hypotheses. With respect to our view that the anxiety-ERN relationship reflects reductions in proactive control and compensatory increases in reactive control, one treatment possibility is to train anxious individuals to adopt more of a proactive control strategy. Proactive control training has been successfully implemented in individuals with schizophrenia, resulting in decreased symptoms and more proactive brain activity (Edwards et al., 2010), as well as in older adults who tend to engage in reactive control strategies before, but not after, training (Braver et al., 2009; Czernochowski et al., 2010; Jimura and Braver, 2010). We predict that proactive control training in worriers would result in reductions in ERN magnitude that might also mediate the effectiveness of the intervention in terms of symptom reduction. Similarly, another possibility for testing our hypothesis comes from Ramirez and Beilock's (2011) recent demonstration that emotional expressive writing improves test performance in high test anxious individuals via its effects on reducing worries and freeing up proactive resources for active goal maintenance. We expect that expressive writing about worries would likewise result in reduced ERN magnitude in highly apprehensive individuals.

A particularly exciting feature of this last set of predictions concerning treatment effects on the ERN in anxious individuals is that it provides a context in which to interpret broader effects of anxiety treatment on the ERN. To date, one study in pediatric OCD patients showed that the ERN did not change with successful cognitive-behavioral treatment (CBT) of OCD (Hajcak et al., 2008). This study has been cited as evidence for a “trait” biomarker or “endophenotype” interpretation of enhanced ERN in anxiety (e.g., Olvet and Hajcak, 2008). However, there seem to be three problems with this conclusion: (1) despite symptom reduction in the OCD patients, post-treatment scores still placed them around the clinical cutoff for an OCD diagnosis, (2) CBT is an intervention designed to reduce anxiety symptoms, not alter underlying neural mechanism involved in cognitive control (i.e., ERN), and (3) the study was conducted in children and adolescents for whom the anxiety-ERN relationship may be different than in adults (Meyer et al., 2012). In this way, even though patients showed reduced OCD symptoms after treatment, they still demonstrated anxiety-related compensatory effort, as reflected in enhanced ERN. The focus of our predictions is not on reducing anxiety symptoms *per se*, but rather to change the functional relationship between worry and cognitive functioning (cf. Ramirez and Beilock, 2011). For instance, the purpose of the expressive writing intervention is to target the *mechanism* involved in anxiety's effects on cognition. This approach will not only help test our predictions set forth here but it may also inform treatments of anxiety and their impact on performance.

The current framework provides an important link between anxiety research and computational models of cognition. Thus, we suggest that future research in this area (and in other allied areas as well) apply computational modeling to test predictions about the associations between anxiety and error-monitoring ERPs and related performance measures. Yeung and Cohen (2006), for instance, demonstrated the power of applying computational modeling to understand ACC-mediated monitoring deficits in lesion patients. Interestingly, they showed that reduced ERN in patients with ACC lesions could be modeled as resulting from impaired attention control rather than specific impairments in conflict-monitoring *per se*. Applying this modeling technique to the anxiety-ERN relationship, in particular by implementing distinct proactive and reactive control modes in a single model (e.g., De Pisapia and Braver, 2006), represents an exciting direction for future research. This approach might help illuminate whether anxiety affects ACC-mediated monitoring functions directly, as envisioned in current theories that emphasize tight linkages between control and affective functions in ACC (e.g., Shackman et al., 2011; Hajcak, 2012), or rather has an indirect impact through its effects on cognitive control modes (e.g., Braver, 2012), as suggested by our analysis.

This framework also provides the foundation for incorporating other conflict- and error-monitoring ERPs that have failed to be adequately addressed by researchers primarily interested in the anxiety-ERN relationship. Regarding the CRN, for example, the results of the current meta-analysis suggest that it is not reliably associated with anxiety, thus failing to support the notion of general overactive action monitoring in anxiety (e.g., Hajcak et al., 2003; Endrass et al., 2008). The error positivity (Pe)—a centro-parietally maximal ERP that follows the ERN (See Figure 1; Falkenstein et al., 2000)—is another error-monitoring ERP that has received limited attention in the anxiety literature. The Pe appears to index explicit error-related processing, including the detection and signaling of errors (Yeung and Summerfield, 2012). To date, research is equivocal, with some studies showing reduced Pe (Moser et al., 2012), some showing enhanced Pe (Weinberg et al., 2010) and still others showing no association (Weinberg et al., 2012a) in anxiety. Again, such inconsistent findings argue against a general impairment in error/action monitoring.

The N2, a fronto-central negativity observed around 250–350 ms in the stimulus-locked ERP on correct trials, is a relevant action-monitoring ERP that is purported to reflect pre-response conflict elicited by the co-activation of correct and incorrect responses when stimuli are associated with both (e.g., incongruent flanker stimuli; Yeung et al., 2004). Unfortunately, the N2 is even more ignored than the Pe in anxiety research. Two studies, not included in the current analysis because they did not report ERN data, however, suggest enhanced N2 in trait anxious college students (Righi et al., 2009; Sehlmeyer et al., 2010). If enhanced N2 were to emerge as a reliable marker of anxiety in future studies, it would suggest a more general effect of anxiety on conflict monitoring (Yeung et al., 2004).

### Related accounts of enhanced ERN in anxiety

The major advance of our proposal is that it attempts to directly account for the relationship between anxiety and the ERN. Although there exist emotional-motivational accounts of the ERN and its within- and between-subjects variation (Pailing and Segalowitz, 2004; Weinberg et al., 2012b), none make specific predictions about the relationship between anxiety and the ERN. Rather, existing accounts are much broader in their assertions regarding the functional significance of the ERN and its variation across individuals. Nonetheless, to the extent that existing emotional-motivational accounts can be applied to the anxiety-ERN relationship, we next address how they fare with regard to their ability to explain existing data.

Researchers have suggested that the ERN is an affective or emotional response to errors (Luu and Tucker, 2004; Pailing and Segalowitz, 2004), in large part because of associations noted between the ERN and individual differences in emotional traits like anxiety. According to this view, then, an enhanced ERN in anxious individuals reflects their heightened negative emotional response to or concerns over mistakes (Bush et al., 2000; Gehring and Willoughby, 2002; Hajcak et al., 2005). Many earlier studies pointed to both heightened ERN amplitude and overactive error-related ACC activity in anxiety as evidence of a dysfunctional affective response to errors, particularly in individuals with OCD (Gehring et al., 2000; Johannes et al., 2001). Functional imaging evidence showing *rostral* ACC enhancement in response to errors in OCD patients (Fitzgerald et al., 2005) was considered strong support for this claim, as the rostral subdivision is often considered the “affective/emotional” portion of ACC, as opposed to the “cognitive” subdivision that lies dorsally (Bush et al., 2000).

A related conceptualization suggests that variation in the magnitude of the ERN reflects individual differences in defensive reactivity (Hajcak and Foti, 2008; Hajcak, 2012; Weinberg et al., 2012a). That is, the ERN carries information aimed at mobilizing resources to protect the organism against subsequent negative events, with this response being sensitive to individual differences in aversiveness of errors. These authors situate the ERN in a broader network of defensive motivational systems involved in executing a cascade of physiological, cognitive, and behavioral responses when potential threats are detected (Lang et al., 1997; Bradley et al., 2001; Bradley, 2008). In this view, the ERN is a neural marker of a broader neurobehavioral trait—that is, a stable individual difference with identifiable referents in neurobiology and behavior (Patrick and Bernat, 2010; Patrick et al., 2012)—of defensive reactivity. Anxiety is included in this model as reflecting individual differences in defensive reactivity thereby supporting the theory's primary contention.

Although the affective response and defensive reactivity models provide plausible accounts of heightened ERN amplitude in anxiety, they only loosely address the fact that some forms of anxiety are more closely tied to enhanced ERN than others. Our conceptual framework, on the other hand, uses this distinction as foundational for specifying the relationship between anxiety and the ERN. There are also contradictory findings in the literature that point to additional weaknesses in current approaches to conceptualizing the connection between anxiety and the ERN.

With regard to the affective response interpretation, the cognitive vs. affective subdivision model of the ACC is not supported by extant research (Shackman et al., 2011). Thus, it is unclear whether enhanced rostral ACC activation following errors in anxious individuals is indicative of an affective response *per se* (cf. Poldrack, 2011 for problems with reverse inference in general). Rather, as Shackman et al. suggest, such ACC activation in anxious individuals may reflect a more domain general “adaptive control” response. Moreover, modulations of ACC activity should not be conflated with those of the ERN given the potential for multiple sources to contribute to the generation of the ERN (Gehring et al., 2012). Evidence from our own work further demonstrates this point. Specifically, although ACC activity is enhanced during symptom provocation in simple phobics (e.g., spider phobics; Rauch et al., 1995), we showed that the ERN is not (Moser et al., 2005).

Regarding the defensive reactivity interpretation, evidence speaking directly to the assertion that “… anxious individuals who are characterized by increased ERNs may exhibit a greater defensive response to errors compared with non-anxious individuals” (Hajcak and Foti, 2008, p. 106) is lacking. In fact, Endrass and colleagues' (2010) failure to show modulation of the ERN by punishment in an OCD sample is inconsistent with a defensive reactivity account. If enhanced ERN in anxiety reflects the aversiveness of errors, it stands to reason that the ERN should have been enhanced during the punishment condition in the OCD sample. That this result was not observed suggests the aversiveness of the error did not significantly contribute to enhanced ERN in the OCD sample in either the baseline or punishment condition. Riesel et al. (2012), on the other hand, did find that punishment enhanced the ERN in high trait anxious individuals but not low trait anxious individuals. However, the authors utilized the STAI-T, which we have shown here is not reliably associated with enhanced ERN. Indeed, high STAI-T individuals in the Riesel et al. study did not show enhanced ERN in the control condition, only a larger enhancement of the ERN from the control to punishment condition. Taken together, extant data are equivocal as to the ability of the defensive reactivity account to explain enhanced ERN in anxiety.

## Concluding remarks

Our overarching goal in this paper has been to provide a foundation for future research addressing the relationship between anxiety and error processing, both quantitatively and conceptually. In particular, we provide estimates of the effect sizes concerning associations between dimensions of anxiety and error-monitoring ERPs elicited in standard conflict tasks. This meta-analytic result provides a more exact understanding of the previous literature and can serve to help researchers design better studies for the future with an eye toward statistical power and precision. We have also articulated a framework that focuses on what enhanced ERN reflects about cognitive dysfunction in anxiety. Our view is that enhanced ERN in anxiety indexes the impact of anxious apprehension—i.e., worry—on post-decisional response conflict by way of its negative influence on active goal maintenance mechanisms and a resulting compensatory increase in “as-needed” reactive control. Such a dynamic reflects what Berggren and Derakshan (2013) call the “hidden cost” of anxiety. As has been suggested, under simple task conditions, this compensatory effort allows anxious individuals to perform as well as non-anxious individuals. Unfortunately, compensatory effects can break down when tasks become more difficult. That is, enhanced ERN provides an index of how hard a worried mind has to work to complete even simple tasks. It can serve as a harbinger of struggle and potential failure on more complex tasks and presumably real-world adaptation. Indeed, the constant distraction and compensatory re-focus is illustrative of how anxiety, and worry, in particular, can drain resources and lead to functional disability.

In sum, we hope this model and our initial ideas for future research represents just the beginning of a deeper understanding of what error- and conflict-related ERPs can tell us about the impact of anxiety on cognition. The promise of more formalized models of cognitive dysfunction in anxiety will be realized to the extent that they offer new insights into how better to identify and treat the world's most ubiquitous mental health problem.

### Conflict of interest statement

The authors declare that the research was conducted in the absence of any commercial or financial relationships that could be construed as a potential conflict of interest.

## Footnotes

^1^It is important to note that all of these studies examined the relationship between anxiety and the response-locked ERN, as previously defined. The negative going ERP component elicited after negative feedback (i.e., feedback-related negativity or FRN; Miltner et al., 1997) has been less consistently linked to anxiety. In fact, some studies have noted attenuated FRN amplitudes in anxiety (Gu et al., 2010; Aarts and Pourtois, 2012; O'Toole et al., 2012 see Simons, 2010 for a review). Given that the majority of the anxiety research has examined the response-locked ERN, this component will constitute the focus of the present investigation.

^2^See Appendix for converging findings using a random effects model.

^3^In a sample of over 500 undergraduates, PSWQ was more than three times as highly correlated with BIS (*r* = 0.65, *p* < 0.001, *n* = 531) than was MASQ-AA with BIS (*r* = 0.21, *p* < 0.001, *n* = 526).

^4^ We thank Editor Alex Shackman for pointing out this potential confound.

^5^With respect to the ΔERN, it is important to note that it includes variance accounted for by the ERN and it is therefore difficult to discern whether its association with anxiety is driven by variance attributable to the ERN itself. Multivariate analyses are necessary to address this issue in future studies.

## Appendix

Given that the varying-coefficient model—the basis for the analysis presented in the main text—has rarely been applied in the published literature, we also present results computed from a more common meta-analytic framework. As these studies were rather heterogeneous in their reported effect sizes, our second analysis was conducted within the context of a random effects model (Cumming, 2012). Point estimates, 95% CIs and heterogeneity statistics were computed using Comprehensive Meta-Analysis software (v.2; Borenstein et al., 2005).

The results of this analysis are presented in Table A1. Overall, these results closely mirror the findings from the main analysis. As in the main analysis, both the ERN and ΔERN showed significant associations with measures of anxiety. Importantly, analyses of the heterogeneity between data sets revealed that effect sizes were significantly larger in studies examining anxious apprehension compared to mixed anxiety for the both the ERN and ΔERN. The results of this analysis diverge from those presented in the text in two, relatively minor, ways: first, mixed anxiety no longer shows a significant association with either the ERN or ΔERN. Second, the CRN now shows a small, but significant, association with anxiety. As before, the CRN does not show moderation by anxiety dimension group.

**Table A1:** **Results from the Meta-Analysis using the random effects model**.

**Sample**	***r***	**Lower limit**	**Upper limit**	***Q***	***p***
**ERN**					
Overall^†^	−**0.254**	−**0.331**	−**0.173**	8.95	0.003
Apprehension	−**0.329**	−**0.411**	−**0.241**	–	–
Mixed	−0.110	−0.224	0.007	–	–
**CRN**					
Overall	−**0.059**	−**0.115**	−**0.002**	0.88	0.358
**ΔERN**					
Overall	−**0.195**	−**0.273**	−**0.115**	13.05	<0.001
Apprehension	−**0.275**	−**0.350**	−**0.197**	–	–
Mixed	−0.043	−0.142	0.056	–	–

Key:

*r: aggregate effect size of association with anxiety*.

*Lower Limit/Upper Limit: The bounds for the 95% confidence intervals for the aggregate correlation (bold type indicates that the confidence interval does not include 0)*.

*Q: The heterogeneity statistic used to test for moderation between Anxious Apprehension and Mixed anxiety*.

*p: Significance for the Q statistic. Both the ERN and ΔERN continue to show significant moderation after adjusting for three comparisons*.

† *As before, we first conducted this analysis without the anxious arousal data from Moran et al. (2012). When these data an included, both the ERN (r = −0.247; 95% CIs: −0.321; −0.169) and ΔERN (r = −0.191; 95% CIs: −0.264; −0.116) continued to show significant associations with anxiety. The CRN, however, did not (r = −0.046; 95% CIs: −0.099; 0.008)*.
